# The relationship between vitamin D receptor gene polymorphism and deciduous tooth decay in Chinese children

**DOI:** 10.1186/s12903-017-0398-x

**Published:** 2017-07-11

**Authors:** Yuan-yuan Kong, Jian-mao Zheng, Wen-juan Zhang, Qian-zhou Jiang, Xue-chao Yang, Miao Yu, Su-juan Zeng

**Affiliations:** 1 Key Laboratory of Oral Medicine, Guangzhou Institute of Oral Disease, Stomatology Hospital of Guangzhou Medical University, 39# Huangsha Road, Guangzhou, 510140 China; 2 Department of Endodontics, Stomatology Hospital of Guangzhou Medical University, Guangzhou, 510140 China; 3 Guangdong Provincial Key Laboratory of Stomatology, Department of Operative Dentistry and Endodontics, Guanghua School of Stomatology, Affiliated Stomatological Hospital, Sun Yat-sen University, Guangzhou, 510140 China; 4 Department of Oral Digital Center, Stomatology Hospital of Guangzhou Medical University, Guangzhou, 510140 China; 5 Department of Dongfengxi Clinic, Stomatology Hospital of Guangzhou Medical University, Guangzhou, 510140 China; 6 Department of Pediatric Dentistry, Stomatology Hospital of Guangzhou Medical University, Guangzhou, 510140 China

**Keywords:** Deciduous tooth decay, Vitamin D receptor, Gene polymorphisms

## Abstract

**Background:**

In the present study, we explored the link between vitamin D receptor (VDR) *Bsm*I, *Taq*I, *Apa*I and *Fok*I gene polymorphisms with deciduous tooth decay in Chinese children.

**Methods:**

Our study included 380 Chinese children aged 4–7 years, whose DNA sample was collected from the buccal mucosa. VDR gene polymorphisms was determined by PCR-RFLP.

**Results:**

The adjusted logistic regression analysis demonstrated that *Bsm*I containing the Bb genotype was linked with the increased risk of deciduous tooth decay (OR = 1.856, 95% CI = [1.184, 2.908], *p* = 0.007). However, VDR polymorphisms *Apa*I, *Taq*I and *Fok*I were not associated with deciduous tooth decay (*Apa*I: OR = 0.839, 95% CI = [0.614, 1.145], *p* = 0.268; *Taq*I: OR = 1.150, 95% CI = [0.495, 2.672], *p* = 0.744; *Fok*I: OR = 0.856, 95% CI = [0.616, 1.191], *p* = 0.356).

**Conclusions:**

Our results showed that VDR *Bsm*I polymorphism was associated with the risk of deciduous tooth decay in Chinese children aged 4–7 years. However, the specific mechanism remains to further verify through experiment.

## Background

Dental caries is one of the most common diseases and its prevalence is more than 50% in many countries [1]. Untreated dental caries can cause further serious problems, such as caries related pulpitis, abscesses, pain, tooth loss and other co-morbidities, which affects growth and well-being in preschool children [2, 3]. As we known, dental caries is a multi-factorial disease, which is caused by environmental factors, behavioral factors and gene-by-environment interactions [4]. Although the importance of genetic factors have been recognized, until now only a few caries susceptibility genes have been identified and verified [5–7].

As a biological macromolecule in nucleus, the VDR gene has the effect of modulating the biological function of the major metabolites of vitamin D, which plays an important role in the formation of teeth, particularly in the calcification of enamel and dentin [8, 9]. Vitamin D is pivotal to maintaining a stable relationship between phosphate and calcium ions, which are known as vital factors in protecting and strengthening the teeth. A randomized trial have found that lack of vitamin D during pregnancy is associated with enamel dysplasia in children [10]. Further cohort study found that enamel dysplasia was associated with risk of dental caries [11, 12]. Vitamin D is not only involved in the regulation of calcium and phosphate levels, but also an important part of the immune system [13]. Vitamin D deficiency leads to uncontrolled changes in the immune system, which then blocks the correct immune response to oral microbial infections that occur during periodontitis or untreated caries [14]. Meta-analysis found that supplemental vitamin D was associated with 47% reduced risk of dental caries [9]. The biological function of Vitamin D is modulated by VDR protein, through interaction with the VDR protein [15]. As reported, the activity of VDR protein is affected by VDR gene polymorphisms [16]. We speculated that VDR polymorphisms might be a genetic factor for deciduous tooth decay. Thus, we performed a case-control study to clarify whether there was association between four VDR gene polymorphisms *Bsm*I, *Taq*I, *Apa*I and *Fok*I with deciduous tooth decay in Chinese children.

## Methods

### Study populations

The study group consisted of 380 Chinese children (203 girls and 177 boys) aged from 4 to 7 years living in the city of Guangzhou, southern China. Children were recruited from the five kindergartens and two primary schools of Liwan district in Guangzhou city. Inclusion criteria involved agreement to comply with the study visits and procedures. The exclusion criteria were according to published study performed by Antunes et al. [17].

All individuals underwent dental examination by two dentists with uniform training at teacher’s office of the kindergarten. Deciduous tooth decay was diagnosed by visual examination and confirmed using a modified protocol recommended by World Health Organization [18]. All examination results were used to calculate the Kappa value, which used to determine the inter-examiner’s reproducibility were 1.

The decayed, missing and filled teeth (dmft) index was used to assess deciduous tooth decay [19]. According to dmft, all individuals were separated into two groups based on caries experience. The results were indicated in the standardized checklist marked with a continuous reference number. The study was approved by the Institutional Ethical Committee of Guangzhou Medical University. All procedures were performed with adequate understanding and written consent of the parents/guardians.

### *S. mutans* infection detection

Salivary tests conducted included information regarding bacteriological counts to quantify *Mutans streptococci* counts. Dentocult SM Strip *mutans* (Orion Diagnostica Oy, Finland) was used to determine the presence of *S. mutans* in saliva of the subjects. Specimen collections were followed by the manufacturer’s instruction. The results were recorded by a classification of 0, 1, 2, 3 according to the manufacturer’s instructions.

### DNA isolation

DNAs were obtained from epithelial cells in buccal mucosa and were extracted with TIANamp Swab DNA Kits (TIANGEN BIOTECH, BEIJING, China) according to the manufacturer’s instructions, then stored at −20 °C [20].

### Identification of *Bsm*I, *Taq*I, *Apa*I and *Fok*I polymorphisms of the VDR gene

The following primer pair was used for polymerase chain reaction amplification of genomic DNA samples: *Bsm*I (5′ –ATA CCT ACT TTG CTG GTT TGC-3’and 3′- AGC CCA TCT CCA TTC CTT G-5′), *Taq*I and *Apa*I (5′ -AGC AGA GCA GAG TTC CAA GCA GA-3’and 3′- ATC TTG GCA TAG AGC AGG TGG CT-5′), *Fok*I (5′ –AGC TGG CCC TGG CAC TGA CTC TGG CTC-3’and 3′- ATG GAA ACA CCT TGC TTC TTC TCC CTC-5′). The process of polymerase chain reaction was referred to published study [21].

The restriction fragment length polymorphism technique was performed in a final reaction volume of 20 μL reaction mixture, containing 0.5 μL *Bsm*I*, Taq*I*, Apa*I *and Fok*I (New England Biolabs, Beijing, China), 5 μL aliquot of polymerase chain reaction products and 14.5 μL ddH_2_O. The BsmI, TaqI, ApaI and FokI fragments were digested by restriction endonuclease. The digestion products of four VDR gene polymorphisms were separated, stained, and recorded under UV light (Transiluminator 4000; Stratagene La Jolla, CA, USA).

### Statistical analysis

Data analysis was performed by the SPSS 20.0 software platform. Odds ratio (OR) calculations and χ^2^ test were performed to evaluate differences of allele, genotype, gender, age and *S. mutans* between caries-experience groups with control groups. *p* < 0.05 was indicated statistically significant difference. The binary logistic regression analysis was performed after adjusting by genotype, age and *S. mutans* factor. The deviation from Hardy-Weinberg equilibrium was tested by standard Chi-square test. Power calculation was analyzed using the Power Analysis and Sample Size (PASS, version 2008). In the present study, 249 cases and 131 controls can achieve a statistical power of 0.8123 to calculate the risk probability of Bb genotype verus bb, which resulting in an OR of 1.845.

## Results

A total of 380 children recruited in this study, 131 (34.47%) were caries-free. The mean age was 5.85 years in caries-free subjects and 5.72 years in caries experience subjects (dmft: 6.6 ± 4.4). A significant difference for factors like age and *S. mutans* between caries experience and caries-free were observed in the present study. The demographic and clinical details of the subjects were presented in Table 1.

**Table 1:** Demographic data and risk factors for caries in the study subjects (*n* = 380)

	Caries experience	Caries-free	*P* value
	(*n* = 249)	(*n* = 131)	
Gender			
Female	131 (52.6%)	72 (55.0%)	0.067
Male	118 (47.4%)	59 (45.0%)	
Mean Age	5.72 ± 0.58	5.85 ± 0.62	0.045
S. *mutans*			
0!	95 (38.2%)	89 (67.9%)	
1!	62 (24.9%)	25 (19.1%)	<0.001
2!	48 (19.3%)	11 (8.4%)	
3!	44 (17.7%)	6 (4.6%)	

X2 test, *p* < 0.05 indicates statistical significance

The genotypes and allele frequency distribution of four polymorphic sites in genes selected amongst caries-free subjects and subjects with caries experience were presented in Table 2. All SNPs were in Hardy-Weinberg equilibrium in unaffected individuals. The variant genotypes and allele frequency of *Bsm*I were different between caries experience and caries-free groups (*p* = 0.005 and *p* = 0.027 respectively). However, no difference was found in genotypes and allele frequency of the polymorphism *Taq*I, *Apa*I, *Fok*I between caries-affected and caries-free individuals (Table 2).

**Table 2:** Allele and genotype frequencies of four VDR gene polymorphisms in 4 ~ 7 years old children

VDR Gene Polymorphisms	Caries experience		Caries-free	*p* value	OR (95% CI)
(rs number)	(*n* = 249)		(*n* = 131)		
*Bsm*I (rs1544410)	BB	0	0	0.005	1.854 (1.209–2.844)
	Bb	152 (61.0%)	60 (45.8%)		
	bb	97 (39.0%)	71 (54.2%)		
	B	152 (30.5%)	60 (22.9%)	0.027	1.479 (1.047–2.090)
	b	346 (69.5%)	202 (77.1%)		
*Taq*I (rs731236)	TT	230 (92.4%)	120 (91.6%)	0.842	1.110 (0.511–2.408)
	Tt	19 (7.6%)	11 (8.4%)		
	tt	0	0		
	T	479 (96.2%)	251 (95.8%)	0.845	1.105 (0.518–2.358)
	t	19 (3.8%)	11 (4.2%)		
*Apa*I (rs7975232)	AA	44 (17.7%)	18 (13.1%)	0.459	
	Aa	87 (34.9%)	43 (33.1%)		
	aa	118 (47.4%)	70 (53.8%)		
	A	175 (35.1%)	79 (30.2%)	0.17	1.255 (0.910–1.731)
	a	323 (64.9%)	183 (69.8%)		
*Fok*I (rs10735810)	FF	69 (27.7%)	34 (26.0%)	0.320	
	Ff	132 (53.0%)	63 (48.0%)		
	ff	48 (19.3%)	34 (26.0%)		
	F	270 (54.2%)	131 (50.0%)	0.285	1.184 (0.878–1.598)
	f	228 (35.8%)	131 (50.0%)		

*P* < 0.05 indicates statistical significance

A binary logistic regression adjusted for genotypes, age and *S. mutans* factor was present in Table 3. In *Bsm*I, the genotype Bb showed significant association with deciduous tooth decay (*p* = 0.007), demonstrating a increased risk for caries (OR = 1.856, 95% CI = [1.184, 2.908]). However, VDR polymorphisms *Apa*I, *Taq*I and *Fok*I were not associated with deciduous tooth decay (*Apa*I: OR = 0.839, 95% CI = [0.614, 1.145], *p* = 0.268; *Taq*I: OR = 1.150, 95% CI = [0.495, 2.672], *p* = 0.744; *Fok*I: OR = 0.856, 95% CI = [0.616, 1.191], *p* = 0.356).

**Table 3:** Binary logistic regression analysis adjusted for genotypes, age and *S. mutans*

Variables	Univariate analysis	
	*p* value	OR (95% CI)
*Bsm*I		
BB		
Bb	0.007	1.856 (1.184–2.908)
bb	reference	
*Taq*I		
TT	reference	
Tt	0.744	1.150 (0.495–2.672)
tt		
*Apa*I		
AA	reference	
Aa	0.268	0.839 (0.614–1.145)
aa		
*Fok*I		
FF	reference	
Ff	0.356	0.856 (0.616–1.191)
ff		
S. *mutans*	reference	
0!	0.658	
1!	0.003	2.330 (1.341–4.051)
2!	<0.001	4.107 (1.994–8.456)
3!	<0.001	6.842 (2.765–16.932)
age	0.153	

*p* < 0.05 indicates statistical significance

## Discussion

Dental caries is caused by a complex interaction between genetics and the environment [22]. Reports of families and animal breeding, together with genomics, have indicated that genetic component was associated with dental caries [23–25], and more than 40% of caries risk was attributed to genetic factors [26]. Published studies found that the caries risk may be determined by patients’ DNA, in addition to environmental factors related to the risk of dental caries, such as oral hygiene, diet, bacteria and host factors [4, 27, 28].

Mutations in genes involved in enamel formation, such as enamelin (ENAM), amelogenin (AMELX), matrix metalloproteinase-20 (MMP-20) and kallikrein-related peptidase (KLK4), were associated with amelogenesis imperfecta [29]. Moreover, studies have found the link between dental caries and amelogenesis imperfecta. Some studies have clarified the genetic variation in AMELX linked with enamel formation contributed to caries susceptibility [30, 31]. Also, genetic variation in MMP-13 and MMP-20 was involved in increased caries risk during the enamel development [28, 32]. The VDR gene, identified as a candidate gene related to dental caries, mediated the biological function of the major metabolite of vitamin D, which was associated with enamel formation [8, 9]. Mutations in VDR gene influenced the activity of VDR protein, which then affected the biological function of Vitamin D [15, 16]. Vitamin D regulates the metabolism of calcium and promotes calcium depositing onto the enamel. A meta-analysis indicated that supplemental vitamin D was linked with more than 40% reduced risk of dental caries [9]. Purvis et al. [10] found that there was relationship between vitamin D deficiency and enamel hypoplasias, which was in turn associated with increased caries risk [11, 12].

In this study, we performed a case-control study to clarify the association of four VDR gene polymorphisms *Bsm*I, *Taq*I, *Apa*I and *Fok*I with deciduous tooth decay in Chinese children. The results showed VDR polymorphisms *Bsm*I containing the Bb genotype was associated with increased risk of deciduous tooth decay (OR = 1.856, 95% CI = [1.184, 2.908], *p* = 0.007). In VDR gene, the SNP *Bsm*I was situated at the 3’UTR corresponding genomic DNA region [33]. However, the molecular mechanisms of deciduous tooth decay regulated by the VDR gene were not certain. We speculated that the SNP *Bsm*I mutation (Bb) at 3’UTR region would affect the mRNA stability, consequently would affect the amount and activity of protein translated. Valdivielso et al. [16] found that the activity of VDR protein was influenced by mutations in the VDR gene. VDR protein mediated the biological function of Vitamin D, via interaction with the Vitamin D, which was associated with dental caries [9, 10, 15].

In contrast, we found VDR polymorphisms *Apa*I, *Taq*I and *Fok*I were not associated with deciduous tooth decay. These results were inconsistent with previous study performed by Hu et al. [34], which determined significant association between VDR polymorphism *Taq*I and permanent tooth decay susceptibility in Chinese adults. According to studies conducted by Bayram M et al. [35] and Borilova Linhartova et al. [36], influences of genetic factors on enamel caries differ between the primary and permanent teeth. Thus, the inconsistency between our results and other reported study may be caused by difference between the primary and permanent dentitions. Cogulu et al. [37] found *Taq*I genotypes in VDR gene might be used as a marker for determining dental caries susceptibility in Turkish children, however our study and other study conducted by Izakovicova Holla et al. [38] found no association of *Taq*I VDR polymorphism with caries, this difference may reflect the geographic and ethnic variations. The frequencies of VDR SNPs in our study (performed in Guangzhou, southern China) were different with the frequencies found within other study by Li et al. (performed in Nanjing, eastern China) [21], for example in *Bsm*I the frequency in our study highly differed with Li’s study, we speculated it was caused by geographic variations (Guangzhou versus Nanjing).

The limitation of this study was that we only investigated the relationship between VDR polymorphisms and caries risk of children in Guangzhou, China; however, dental caries was caused by multiple genetic factors and environment. The mechanism of the possible effect of VDR on susceptibility to dental caries remained unclear, further research in people with different ethnic backgrounds should be performed to reveal the mechanism of VDR gene in caries incidence, and clarify its interactions with other susceptibility genes and environmental factors.

## Conclusions

We observed VDR *Bsm*I polymorphism (rs1544410) was associated with deciduous tooth decay in Chinese children aged 4–7 years. However, the specific mechanism remains to further verify through experiment.

## Abbreviations

AMELX: Amelogenin
Dmft: Decayed, missing and filled teeth
ENAM: Enamelin
KLK4: Kallikrein-related peptidase
MMP-20: Matrix metalloproteinase-20
OR: Odds ratio
PCR-RFLP: Polymerase chain reaction-restriction fragment length polymorphism
VDR: Vitamin D receptor
